# Real-time driver identification in IoV: A deep learning and cloud integration approach

**DOI:** 10.1016/j.heliyon.2024.e28109

**Published:** 2024-03-16

**Authors:** Hassan Muwafaq Gheni, Laith A. AbdulRahaim, Abdallah Abdellatif

**Affiliations:** a Electrical Engineering Department, College of Engineering, University of Babylon, Babylon, Iraq; b Computer Techniques Engineering Department, Al-Mustaqbal University College, Babylon, 51001, Iraq; c Department of Electrical Engineering, Faculty of Engineering, Universiti Malaya, Kuala Lumpur, 50603, Malaysia

**Keywords:** Internet of vehicle, Driver identification, Driver behaviour, Cloud computing, Deep learning

## Abstract

The Internet of Vehicles (IoV) emerges as a pivotal extension of the Internet of Things (IoT), specifically geared towards transforming the automotive landscape. In this evolving ecosystem, the demand for a seamless end-to-end system becomes paramount for enhancing operational efficiency and safety. Hence, this study introduces an innovative method for real-time driver identification by integrating cloud computing with deep learning. Utilizing the integrated capabilities of Google Cloud, Thingsboard, and Apache Kafka, the developed solution tailored for IoV technology is adept at managing real-time data collection, processing, prediction, and visualization, with resilience against sensor data anomalies. Also, this research suggests an appropriate method for driver identification by utilizing a combination of Convolutional Neural Networks (CNN) and multi-head self-attention in the proposed approach. The proposed model is validated on two datasets: Security and collected. Moreover, the results show that the proposed model surpassed the previous works by achieving an accuracy and F1 score of 99.95%. Even when challenged with data anomalies, this model maintains a high accuracy of 96.2%. By achieving accurate driver identification results, the proposed end-to-end IoV system can aid in optimizing fleet management, vehicle security, personalized driving experiences, insurance, and risk assessment. This emphasizes its potential for road safety and managing transportation more effectively.

## 1 Introduction

In recent years, the ongoing development of computer and communication technologies has played a significant role in the creation of intelligent transportation. Today, vehicles contain over 50 computer systems managing functions ranging from safety to infotainment. This evolution in car architecture has allowed automotive manufacturers to integrate new technologies, including WIFI, GPS navigation, and 5G connectivity, paving the way for innovative products and cloud-based services. The continued improvement of the Internet of Vehicles (IoV) and communication technology has significantly impacted the evolution of cars [1].

In an environment where vehicles are connected to the cloud, the drivers can access their vehicles linked to a cloud server that offers various driving-related services, including insurance services [2,3]. Nevertheless, the connectivity of vehicles via edge and cloud can increase the risk of vehicle theft [4,5]. To address these issues, research into driver identification has emerged to improve driver profiling and car security [4,[6], [7], [8]]. Characterizing drivers' driving habits can provide valuable insights for various tasks, such as driving style detection, driver drowsiness detection, impaired driving detection, driver identification, driver behaviour modelling, risk assessment, and driving event detection or prediction. These insights can be beneficial for improving the understanding of the factors that influence driving behaviour and developing effective interventions to address safety-related concerns. Therefore, characterizing driving habits can be an essential component of research on driving behaviour and related fields [[9], [10], [11]]. Several driver identification approaches exist, including biometric identification, using sensors for voiceprints, fingerprints, iris, face recognition [[12], [13], [14], [15], [16], [17], [18], [19]], and Smartphone Authentication, using the driver's smartphone sensors [20]. Another promising approach involves gathering driving data via Controller Area Network (CAN-BUS) signals from sensors embedded in the vehicle [21,22]. An accurate system customized for the IoV technology can offer several benefits for driver identification tasks in intelligent transportation [23,24].

Several techniques from the literature were utilized to identify the driver based on the driver's habits and behaviour. Driver identification techniques can be categorized into various categories: handcrafted, machine learning (ML), deep learning (DL), and hybrid methods, based on features obtained from CAN-BUS, smartphone, and Internal measurement unit (IMU) sensor raw data. However, some of them have drawbacks. At the beginning of 2005, Wakita et al. [25] proposed utilizing behavioural cues recorded during car-following tasks for driver identification. Their simulation-based study demonstrated that the manipulation signals used by drivers and a Gaussian mixture model (GMM) could effectively distinguish between different drivers. Miyajima et al. [26] built upon and enhanced Wakita et al.'s model. They introduced a novel approach of incorporating fast Fourier transform (FFT) analysis of brake pedal and accelerator as additional features to enhance the accuracy of driver identification using GMM.

Ezzini et al. [27] conducted a study in which they utilized conventional ML algorithms, including K-Nearest Neighbors (KNN), Random Forest (RF), and Extra Trees. The algorithms were evaluated using diverse processing techniques across various datasets and window sizes, resulting in a favourable cross-validation score. The study identified two distinct categories of features: driving pattern-related and driver-related pattern features, which included physiological measures such as heart rate. Del et al. [28] employed an Artificial Neural Network (ANN) to analyze signals from the CAN-BUS while incorporating cepstral features. A sliding window approach was utilized for data preprocessing, resulting in an 84% accuracy in driver identification. Zhang et al. [29] introduced a new approach for driver classification using a window-based Support Vector Machine (SVM) algorithm. The authors emphasized the significance of data source integration, including phone sensors, car sensors, and the combination of both, in improving classification accuracy. The highest accuracy score of 86.67% was attained through the utilization of combined data sources. Martinez et al. [30] utilized an Extreme Learning Machine (ELM) and employed different techniques for extracting features, such as time and frequency-domain features. They have been derived from the CAN-BUS data. The study achieved a recognition accuracy of 84.36% in identifying 11 different drivers. Rahim et al. [31] presented a method for identifying drivers based on 'zero' and 'stable' events. After feature extraction, the feature vector was fed into SVM, RF, and KNN models. Using the RF technique, the proposed scheme produced the maximum accuracy, with an average identification rate of 95.89% for 25 drivers. In addition, Kwak et al. [32] created feature vectors with Shannon's entropy and wavelet energy entropy as the classifier inputs. On highways, the XGBoost classifier accurately detected drivers with a 91.6% accuracy rate. Kwak et al. [4] proposed four ML algorithms, including Decision Trees, Random Forests, K-Nearest Neighbors, and Multilayer Perceptron, for identifying drivers based on 15 features extracted from the CAN-BUS. The study aimed to develop a model that can accurately identify drivers based on their driving behaviour using ML techniques. Hallac et al. [33] concentrated on detecting turning events, which was done by implementing RF classifier that achieved an accuracy of 76.9%.

Similarly, Enev et al. [6] applied a sliding window preprocessing technique and an RF classifier to analyze time-series CAN log signals. The accuracy score ranged from 87% to 91%, depending on the number of drivers involved. Additionally, the study employed an ELM and various feature extraction techniques, including frequency and time-domain features from CAN-bus data, to achieve an accuracy score of 84.36% in detecting 11 drivers. The selected algorithms were evaluated and compared based on their performance in the driver identification task. Notably, the current methods based on ML have two drawbacks, including requiring manually created features and prior knowledge, feature engineering, and learning algorithms frequently call for step-by-step tuning, which cannot ensure the best outcome.

Recently, there has been a shift from conventional ML techniques to deep learning algorithms for feature extraction and their application in driver identification topics [34]. Jong et al. [35] utilized various techniques, including 1D Convolutional Neural Networks (CNN), specific section extraction normalization, and post-processing to improve driver identification accuracy. The study used real-time raw data from the CAN-BUS to train and test the CNN model, achieving an average accuracy score of 90%. Abenezer et al. [36] introduced a Long Short Term Memory (LSTM) model that utilizes vehicle telematics data to predict the identity of the driver based on their distinctive driving patterns. The study demonstrated a superior level of accuracy compared to conventional methods. Abdennour et al. [37] develop a residual convolution network technique for driver identification, utilizing a CNN architecture and using the input sequence in an overlapping sliding window, without any modifications. The study's experimental findings demonstrated that this approach exhibited superior performance compared to conventional ML algorithms and marginally outperformed combinations of CNN and Recurrent Neural Network (RNN) models.

Hybrid methods in ML were used to hold promise for improving the accuracy and robustness of driver identification systems by leveraging the strengths of different algorithms and techniques. Furthermore, overcomes DL limitations. Shan et al. [38] proposed a method that has been implemented in a container environment using NVIDIA Docker on embedded systems, including Xavier, TX2, and Nano, and extensively evaluated using various performance metrics to gauge its effectiveness and achieve an accuracy 98.5%. Zhang et al. [39] created multiple Deep Learning (DL) architectures that combined Convolutional Neural Network (CNN) and RNN models. These architectures, including DeepConvGRU-Attention, DeepConvLSTM-Attention, and DeepConvGRU, were applied to standardized CAN-bus data that had undergone overlapping sliding window segmentation. As a result, the identification accuracy ranged from 97.72% to 98.36%. El Mekki et al. [40] CNN and RNN, specifically LSTM, were utilized in this study. Additionally, a cross-validation technique was employed to ensure the reproducibility of results when applied to unseen realistic data. The proposed model underwent testing on various datasets and was implemented within the Automotive Grade Linux Framework, serving as a real-time anti-theft and driver profiling system. An ensemble deep learning framework for driver identification that combined a modified one-dimensional convolutional neural network (M 1-D CNN) with bidirectional long short-term memory (BLSTM). Four different data augmentation techniques were employed to address the few-shot learning issue. The proposed approach was evaluated on driver identification tasks and demonstrated promising results [41]. Azadani et al. [42] developed several ML and deep learning models for classification. The models included Random Forest, KNN, Decision Tree, CNN, LSTM, Deep Neural Network (DNN), as well as a hybrid model, DeepConvLSTM, which combined CNN and LSTM, achieving high accuracy, depending on the sliding window and statistical features. Azadani et al. [43] present a methodology for obtaining the latent representation of driving data through unsupervised triplet loss training. A stacked encoder architecture is also constructed using dilated causal convolutions and residual blocks. Hongyu et al. [44] this study collected naturalistic driving data from 20 drivers along a fixed testing route, encompassing various road types and traffic conditions. Abu-gellban et al. [45] proposed a model that employs Gated Recurrent Unit (GRU) and Fully Convolutional Networks (FCN) to capture long and short-term patterns in driving behaviours effectively. To improve training efficiency, the Segmented Feature Generation (SFG) algorithm was utilized to reduce the state space of driving behaviours by segmenting them with a window size for analysis.

In the context of intelligent transportation and Internet of Vehicles (IoV) technology, this study addresses the limitations of existing driver identification systems [23,24]. Current research in this field often relies on complex and computationally intensive models that depend heavily on handcrafted features and lack comprehensive testing for real-time implementation [27,31,33]. Table 1 presents a comprehensive comparison of existing works, providing a detailed overview of the different approaches in the driver identification field.

To address these research gaps, this paper introduces an innovative deep-learning structure that leverages multi-head self-attention mechanisms combined with a CNN-based model. These mechanisms, known for their superior interpretability, adaptability, and capability to capture intricate relationships within data, could potentially revolutionize driver identification tasks. This approach mitigates the need for manual feature engineering and enhances the overall performance of driver identification systems by capturing complex data relationships more effectively. In addition, this work significantly emphasizes real-time implementation by proposing a comprehensive end-to-end cloud-based system tailored for IoV technology. This system seamlessly integrates data collection, processing, prediction, and visualization in real-time, demonstrating its effectiveness and reliability in handling real-world scenarios, including sensor data anomalies. Importantly, this study represents the first to test such a model within an end-to-end cloud-based system, highlighting its novelty and practical implications in the IoV domain. This study presents two key contributions.

1. This study proposes an accurate driver identification technique based on the Convolution Neural Network and multi-head self-attention approach.
2. The development of an integrated cloud-based platform purposed to facilitate real-time driver identification in the IoV realm, converging the multiple stages of the identification process.
3. Utilization of Apache Kafka server for real-time deep learning prediction, specifically in the context of IoT, including OBDII, IMU, and GPS.

## 2 Methodology

This section presents a comprehensive overview of the practical scenarios being considered, which revolve around installing in-vehicle sensors, utilizing data streaming and event processing, real-time data analysis, prediction, and data visualization (see Table 1).

### 2.1 Data collected and preprocessed

#### 2.1.1 Data Acquisition System design and installation

**Fig. .1:**
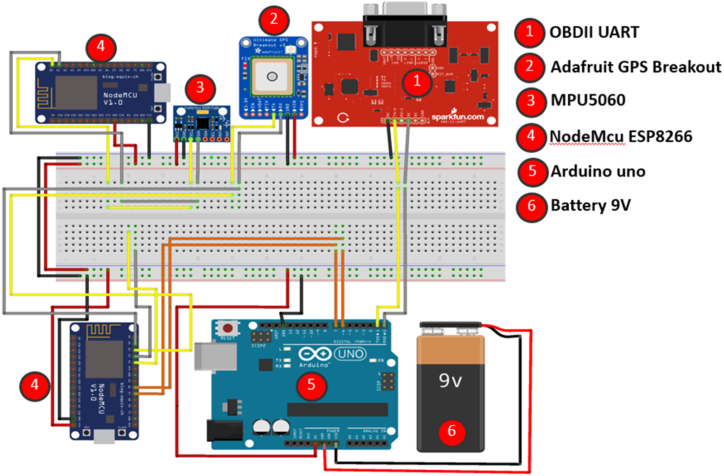
Schematic diagram of the proposed DAS.

The limited availability of publicly accessible naturalistic driving data, particularly those incorporating CAN-BUS-based data, presents a challenge for driver identification research. Therefore, before implementing our driver identification methodology, we conducted an empirical study involving real-world driving scenarios to collect relevant driving data. Fig. 1 presents the schematic design of this research study's proposed Data Acquisition System (DAS). The DAS comprises a comprehensive array of strategically positioned sensors and devices within the vehicle to gather data encompassing various aspects of vehicle performance and driver behaviour. Specifically, the On-Board Diagnostics (OBD-II) reader is typically located beneath the dashboard, the IMU is mounted inside the vehicle's steering mechanism, and the GPS sensor is installed either on the dashboard or the vehicle's roof. These sensors play a crucial role in collecting essential data on acceleration, deceleration, speed, location, fuel consumption, and other pertinent factors. Together, they enable the comprehensive tracking and monitoring of crucial parameters associated with vehicle performance and driver behaviour. Table 2 presents the in-vehicle features collected.

**Table 1:** Related Research on driver identification.

Research	Data used		Model	Deployment	Window size - overlap	Anomaly detection	Number of parameters and floating point (flops)	Statistical Test
	Public	Collected (Features)						
Ezzini et al. [27]	Security data set, UAH, and HCi LAB	**⤬**	K-Nearest Neighbors, Random Forest, Extra Trees	**⤬**	60	**⤬**	**⤬**	**⤬**
Campo et al. [28]	**⤬**	brake pedal pressure and gas pedal pressure	ANN	**⤬**	**⤬**	**⤬**	**⤬**	**⤬**
Zhang et al. [29]	⤬	Throttle position B, Gyroscope Torque GPS Accelerometer, Acc. pedal position E, Engine RPM, Acc. Pedal position D Throttle position manifold, Relative throttle position	SVM	**⤬**	30	**⤬**	**⤬**	**⤬**
Martinez et al. [30]	UYANIK in Turkey	⤬	Extreme-learning machine core	⤬	128	**⤬**	**⤬**	**⤬**
Rahim et al. [31]	Beijing taxis dataset, Beijing Metrobuses Dataset	⤬	SVM	⤬	⤬	**⤬**	**⤬**	**⤬**
Kwak et al. [32]	⤬	Minimum indicated engine torque, Current spark timing, Torque of friction, Steering wheel angle, Intake air pressure, Accelerator pedal value, Fuel consumption, Engine fuel cut-off, Calculated load value, Short term fuel trim bank, Throttle position signal, Engine soaking time, Long term fuel trim bank 1, Vehicle speed, Brake switch ON and OFF, Engine coolant temperature, Engine idle target speed, Flywheel torque, Activation of air compressor, Calculated road gradient, Torque convertor speed, Current gear, Transmission oil temperature, Clutch operation acknowledge, Steering wheel speed, and Yaw rate	wavelet transform, Entropy, and (SVM, Xboost, RF)	⤬	60, 90, 120, and 150	**⤬**	**⤬**	**⤬**
Hallac et al. [33]	⤬	Engine RPM, Gas pedal position, Torque, Steering velocity, Vehicle velocity, Steering wheel angle, Lateral acceleration, Vehicle heading, Brake pedal position, Forward acceleration, Throttle position, Steering acceleration.	RF	⤬	⤬	**⤬**	**⤬**	**⤬**
Jong et al. [35]	⤬	Steering wheel rotation speed, Longitudinal acceleration, Brake pedal pressure, Accelerator pedal pressure, Vehicle Speed, Engine RPM, Lateral acceleration (Lat-Accel), Yawrate, Air blower, Dynamic traction control	CNN	⤬	75	**⤬**	**⤬**	**⤬**
Girma et al. [36]	Security data set, Vehicular data trace Dataset-1, Vehicular data trace Dataset2	⤬	LSTM	⤬	120–60	**⤬**	**⤬**	**⤬**
Abdennour et al. [37]	Security data set,	⤬	RCN	⤬	60–6	**⤬**	**⤬**	
Shan et al. [38]	Security data set	⤬	Hybrid depth-wise CONV-LSTM/GRU	Evaluate the training time of a compressed ML model on three different hardware platforms: NVIDIA Jetson TX2, Jetson Xavier, and Jetson Nano.	40–6	**✓**	**119,832 o.233 million FLOPs**	**⤬**
Zhang et al. [39]	Security data set	⤬	Hybrid FCN-LSTM with self-attention	⤬	60–6	**⤬**	**⤬**	**⤬**
El Mekki et al. [40]	Security data set	⤬	Hybrid FCN-LSTM	implemented in Automotive collab Grade Linux Framework as a real-time anti-theft and driver profiling system	60–6	**✓**	**⤬**	**⤬**
Azadani et al. [42]	Security data set	(i) steering wheel angle speed, (ii) brake pressure, (iii) speed, (iv) longitudinal acceleration, (v) accelerator pedal position, (vi) yaw rate, (vii) engine rotation speed, (viii) lateral acceleration, and (ix) steering wheel angle	Hybrid 1D–CNN–BLSTM	⤬	50–25	**⤬**	**102184 parameter**	**⤬**
Azadani et al. [43]	Security data set	⤬	Hybrid DeepConvLSTM	⤬	90	**⤬**	**⤬**	**⤬**
Hongyu et al. [44]	⤬	(i) steering wheel angle speed, (ii) brake pressure, (iii) speed, (iv) longitudinal acceleration, (v) accelerator pedal position, (vi) yaw rate, (vii) engine rotation speed, (viii) lateral acceleration, and (ix) steering wheel angle	1D-CNN	⤬	5–4	**⤬**	**72365**	**⤬**
Abu-gellban et al. [45]	Security data set	(accelerator pedal), green (filtered accelerator pedal), blue (acceleration speed longitudinal), orange (brake switch), blue-violet (road gradient), dark olive green (acceleration speed lateral), black (steering wheel speed), and magenta (steering wheel angle).	LiveDI	⤬	⤬	**⤬**	**⤬**	**⤬**
**Our proposed**	**Security data set**	**wheel velocity front left hand, accelerator pedal value, throttle position signal coolant_temp, speed, wheel velocity rear left hand, long-term fuel trim bank 1, short-term fuel trim bank 1, engine load, intake_air_pressure, rpm, wheel velocity_front_right hand, steering angle.**	**1D CNN-Attention**	**End-to-end cloud-based system. The system is intended to operate in real-time, including data collection, processing, prediction, and visualization, all executed using cloud resources.**	**40–6**	**✓**	**22,570**	**Friedman Statistical Test**

**Table 2:** Collected feature for the used sensor.

Sensor	Location	Sensor function	Reading range	Unit
OBD-II UART	Under steering wheel	accelerator pedal value	0 to 100	%
		throttle position signal	0 to 100	%
		coolant_temp	−40 to 389	F
		long-term fuel trim bank 1	−25 to 25	%
		short-term fuel trim bank 1	−20 to 20	%
		engine load	0 to 100	%
		intake air pressure	−40 to 389	F
		RPM	–	Revelution per minute
		wheel velocity front left-hand	–	Km/h
		wheel velocity front right-hand	–	Km/h
		wheel velocity rear left-hand	–	Km/h
		speed	–	Km/h
IMU (MPU-6050)	Inside steering wheel	Yaw angle	0 to 360	Degree
Adafruit GPS breakout	Vehicle top	Vehicle Location	–	Logitude and latitude

Table 2 summarizes the key sensors incorporated in the DAS, their locations within the vehicle, their respective functions, the range of values they can measure, and the corresponding units for each measurement. This information highlights the diverse data collected by the sensors, enabling a comprehensive analysis of vehicle performance and behaviour during the research study.

The OBD-II UART interface represents a prevalent communication protocol that establishes connections between OBD-II readers and diverse devices, including Arduino and Nodemcu. This standardized interface enables seamless data exchange and interaction among these devices, promoting efficient integration and interoperability within OBD systems. The OBD-II UART protocol relies on a serial connection mechanism, a fundamental medium for transmitting data. This serial-based communication approach ensures a direct and reliable information exchange between the interconnected devices. The MPU-6050 motion sensor is equipped with six axes of motion detection and is commonly used for measuring a vehicle's steering angle. This research study deliberately positioned the sensor on the steering wheel to capture pertinent motion data. A nodemcu microcontroller was employed to capture the sensor's readings and establish a Wi-Fi connection to transmit the data to the Google Cloud platform to facilitate data collection and transmission.

**Fig. .2:**
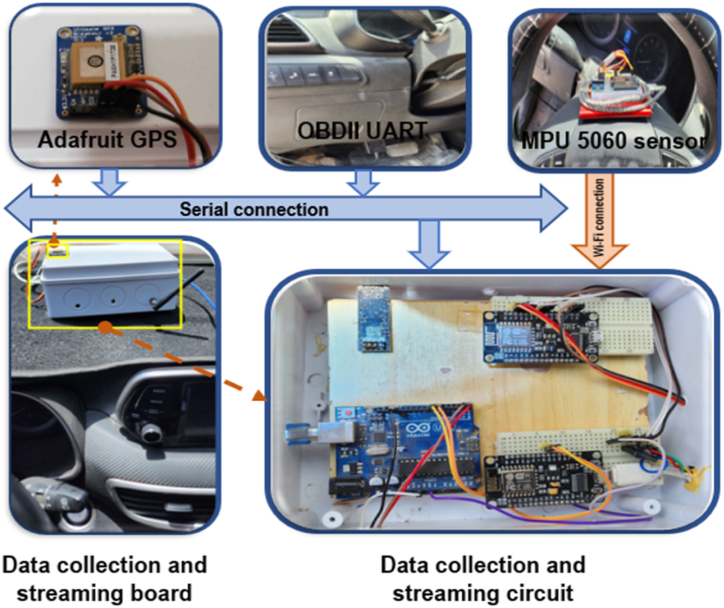
Hardware implementation of the DAS system.

The GPS Adafruit module establishes communication with an Arduino or similar microcontroller using serial communication, enabling real-time tracking and monitoring of the vehicle's position. The GPS module and microcontroller can exchange data seamlessly by utilizing serial communication, facilitating accurate and up-to-date tracking of the vehicle's location. This integration effectively monitors the vehicle's movements and enables applications such as navigation systems, fleet management, and geolocation-based services. The physical setup of a Data Acquisition System (DAS) on a vehicle is depicted in Fig. 2. The system comprises three sensors: the Adafruit GPS breakout, the OBDII UART, and the MPU5060. The communication between the OBDII sensor and the Arduino Uno occurs through a serial connection, while the GPS and MPU5060 sensors communicate with the NodeMCU via a serial connection.

**Fig. .3:**
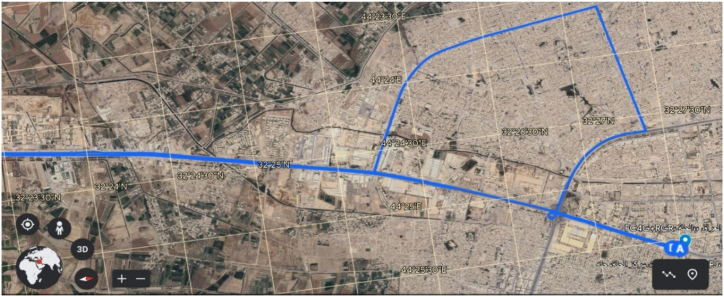
Driving loop location.

**Fig. .4:**
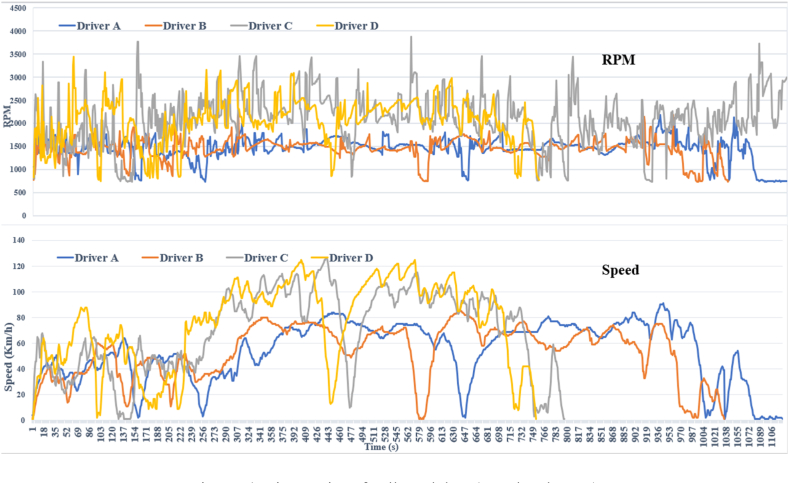
Time series of collected data (speed and RPM).

**Fig. 5:**
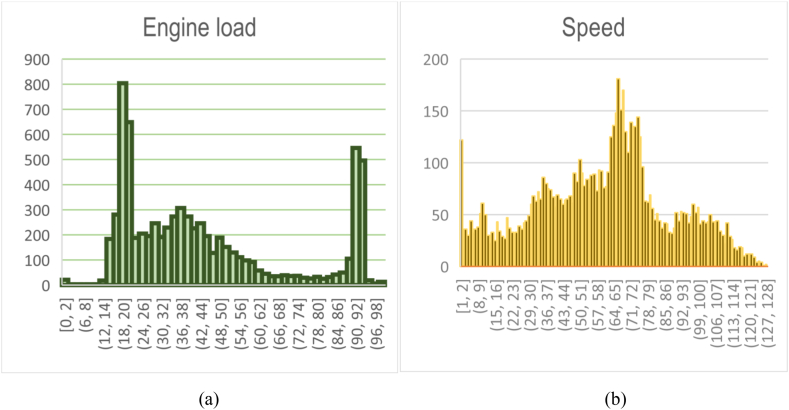
Driving characteristics (a) Engine load (b) Speed.

For the actual vehicle experiment, we recruited four male drivers aged between 20 and 40 years. On average, the participants' ages spanned 28.25 years, with a standard deviation of 6.8 years. Their driving experience ranged from 3 to 15 years, with an average of 6 years and a standard deviation of 4.84 years. The study utilized a recent model from Hyundai Motors Corporation and was conducted in Iraq. Specifically, the experiment took place on a route in Baghdad (BaBIL), which comprised various road types, including city streets, highways, and parking areas, covering 30 km. To maintain consistency, experiments were scheduled between 1 p.m. and 4 p.m. on both weekdays and weekends, starting from February 28, 2022. Each driver completed four round trips on this designated route, ensuring a comprehensive and reliable data collection. Fig. .3, Fig. .4, Fig. 5 present a sample dataset for four drivers, depicting their driving behaviour through RPM and speed distribution profiles. These figures also depict the time-series patterns of CAN data for the participants, highlighting the fluctuations and variations experienced during real-world driving conditions. This version provides a concise and clear overview of the experiment and its methodology.

#### 2.1.2 Feature normalization and sample construction

Data preparation is essential in the DL pipeline between data collection and modelling. Its purpose is to simplify the learning task and expedite the convergence of the DL model. In this study, the collected data consisted of four trips for each driver records spanning approximately 30 min, with measurements captured at 1-s intervals. The data underwent a two-step process to achieve standardization: mean subtraction and division by the standard deviation. Z_Score Normalization was adopted based on its widespread utilization in the machine learning literature. Unlike Min-Max Scaling, Z_Score Normalization considers the data distribution, making it suitable for non-uniformly distributed input variables. The sequential equations below outline the specific steps involved in this data preparation procedure.

(1) $$\mu =\frac{1}{M}\;\sum_{1}^{M}d_{i}$$

(2) $$\sigma =\sqrt{\frac{\sum_{1}^{M}\left(d_{i}-\mu \right)}{M}}$$

(3) $$d_{standardized}=\frac{d_{actual\;}-\;\mu }{\sigma }$$

The parameter μ represents the mean, and σ represents the standard deviation. Additionally, M denotes the size of the dataset, and di represents the value of a datum within the dataset. The process of standardizing the data is presented in Eq. (3).

**Fig. 6:**
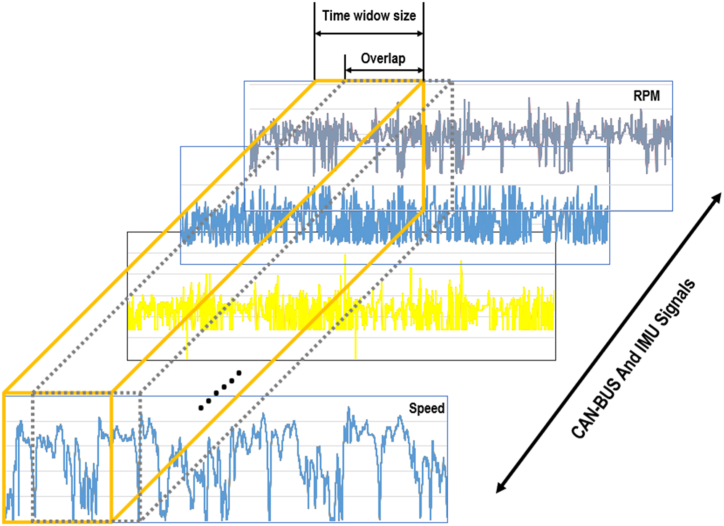
Overlapping windows sliding for 12 features of driver A.

A sliding window approach with specific parameters is employed to preprocess the normalized data for the DL model. The chosen window size was 60 s, implying each window encapsulated 60 s of data. The step size, which designates the interval between successive windows, was determined to be 10 s. As illustrated in Fig. 6, after analyzing one window, the subsequent window shifted by 10 s. This configuration ensured an overlap of data between neighbouring windows, which is crucial for capturing temporal dependencies within the data. This sliding window approach optimized the systematic processing of sequential data by the DL model, thereby enriching its capacity to discern significant patterns and enhance prediction accuracy.

To validate the efficacy of the proposed DL model, we undertook a series of experimental evaluations using publicly accessible CAN-BUS data. Specifically, our study harnessed a dataset encompassing driving data from 10 drivers, each completing two round trips between Korea University and the SANGAM World Cup Stadium. This amounted to roughly 23 h of driving data [4]. For the training phase, driving data from one round trip served as the input, while the subsequent testing phase leveraged data from the alternate round trip. Such a bifurcation in experimental design ensured that the performance of our DL model was assessed under genuine driving scenarios, utilizing authentic driving data spanning a varied driver cohort.

#### 2.1.3 Data streaming and event processing

**Fig. 7:**
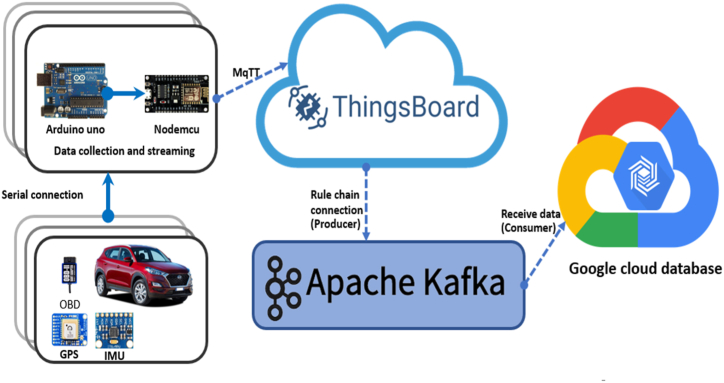
Data streaming from vehicle sensor to Google Cloud.

Data streaming and event processing technologies have recently gained traction as practical tools for driver identification, especially in on-demand services. These technologies can manage continuous data streams related to driver behaviours and patterns. A notable integration between Google Cloud and Thingsboard has been realized in this context. Google Cloud, a reputable cloud computing platform, provides resilient infrastructure and scalable processing capabilities. In contrast, Thingsboard is the dedicated IoT platform for data collection and management, as depicted in Fig. 7.

Data streams from diverse IoT devices such as OBD-II, IMU, and GPS are channelled into Thingsboard and then securely relayed to Google Cloud in real-time. This data is promptly ingested and processed using Apache Kafka, a prominent streaming data processing tool, to accommodate the uninterrupted data flow.

**Fig. 8:**
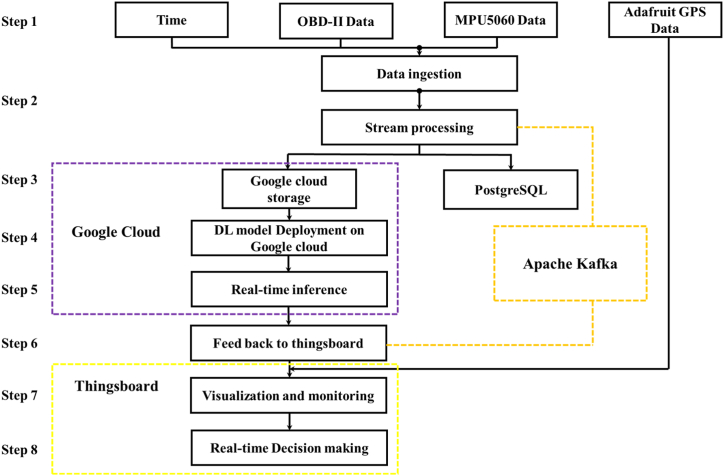
The flowchart of the proposed data stream.

Google Cloud's distributed computing resources host the deep learning models developed with the TensorFlow framework. These models process the streaming data in real-time, enabling immediate inference and decision-making. These models' ensuing predictions or insights are relayed to Thingsboard, paving the way for instantaneous feedback and responsive actions based on the processed data. This integrated ecosystem between Google Cloud and Thingsboard underpins the holistic, real-time deep learning workflow, positioning organizations to harness AI's capabilities on streaming data for prompt and actionable insights. Fig. 8 delineates the successive steps in the data streaming process, from initial data procurement from IoT devices to the visualization of deep learning model outputs on Thingsboard. The following is an overview of the steps involved in the data flow and processing for real-time driver identification through the integration of IoT devices, deep learning models, and unlimited sources of cloud computing.

Step 1 Data Collection: IoT devices acquire data from different sources, including the OBD-II interface, MPU5060 sensor (measuring yaw angle), and Adafruit GPS (capturing longitude and latitude). This data is transmitted to the Thingsboard platform (Refer to Table 2 for details).

Step 2 Data ingestion process: Thingsboard ensures the secure transmission of stream data to Google Cloud. The continuous flow of data is managed by streaming data processing frameworks (Apache Kafka), which handle the data in real-time.

Step 3 Data Storage: Stream data is stored in a suitable data storage system, including Google Cloud Storage and PostgreSQL.

Step 4 Model Deployment: The trained DL model is deployed on Google Cloud, ready for real-time prediction on streaming data.

Step 5 Real-time Inference: The deployed models operate on streaming data to produce real-time predictions.

Step 6 Feedback to Thingsboard: The generated predictions are returned to Thingsboard for real-time feedback and actions.

Step 7 Visualization and Monitoring: The analyzed data and model outputs are visualized and monitored using Thingsboard dashboard tools.

Step 8 Real-time Decision-making: Based on the insights from the deep learning models, real-time actions and decisions can be taken through integrations with other systems or applications.

### 2.2 Multi-head self-attention based on separable 1D-convolution model

The rapid advancement of artificial intelligence has fostered the development of complex deep-learning approaches to address challenging applications, including driver identification. A new model is proposed leveraging the robustness of Separable 1D-CNNs and the flexibility of Multi-Head Self-Attention mechanisms, thereby meticulously addressing the inherent challenges of driver identification. The proposed approach operates in a sequential three-stage process designed to effectively capture the unique driving patterns of individual drivers by processing the time-series driving data. These stages encompass feature extraction using separate 1D convolutional layers, correlation analysis via a multi-head self-attention mechanism, and classification through a fully connected layer.

In the initial stage, we employ three Separate 1D convolutional layers, each followed by a batch normalization (BN) layer and a LeakyReLU activation function. Incorporating separable convolutions capitalizes on their proven computational and parameter efficiency over traditional convolutions. This two-step operation, a depth-wise spatial convolution followed by a pointwise convolution, curtails overfitting while maintaining a robust capability to extract salient features from the input data; the mathematical representation of these two operations is shown in Eq. (4), (5). The LeakyReLU activation function further enhances this stage by preserving data non-linearities and mitigating the "dying ReLU" problem, expressed in Eq. (6).

(4) (I ∗ D) (x, y) = Σ_i Σ_j I(x - i, y - j) *D(i, j)

(5) (I ∗ P)(x, y) = I(x, y) *P

(6) f(x) = max(αx, x) where α = 0.15

Where I denotes the input, D signifies the depth-wise filter, and P is the pointwise filter, as shown in Fig. 10.

The next stage of our methodology introduces the Multi-Head Self-Attention Mechanism, a transformative tool in deep learning. Deriving from the architecture of the transformer model proposed by Ref. [50], this mechanism serves as a strong alternative to traditional recurrent layers, primarily due to its adeptness in handling long-range dependencies, as shown in Fig. 11. The central idea of the self-attention mechanism is its ability to map a query and a set of key-value pairs to an output, where the query, keys, values, and output are all vectors. The output is computed as a weighted sum of the values, where a compatibility function of the query with the corresponding key determines the weight assigned to each value.

The proposed model leverages the concept of multi-head Attention, where the attention function is applied in parallel across different learned linear projections of the queries, keys, and values. This parallel application empowers the model to simultaneously focus on information from different representation subspaces at various positions, a feature impossible with a single attention head. This can be mathematically represented as:

(7) $$\text{Attention}\;\left(Q,K,V\right)=\text{SoftMax}\;\left(\frac{{QK}^{T}}{\sqrt{d_{k}}}\right)V$$

(8) $$\text{MultiHead}\;\left(Q,K,V\right)=\text{Concat}\;\left({head}_{1},\ldots \ldots \ldots ,{head}_{h}\right)\;W^{O}$$

(9) $${head}_{i}=\text{Attention}\;\left(QW_{i}^{Q},KW_{i}^{K},VW_{i}^{V}\right)$$

Where Q, K, and V represent the queries, keys, and values, respectively, and d_k is the dimension of the keys. In equation (9), each Q, K, and V is projected into different representation subspaces using learned linear transformations represented by $QW_{i}^{Q}$, $KW_{i}^{K}$, and $VW_{i}^{V}$, respectively. This mathematical representation of the multi-head self-attention mechanism shows its potential to focus on different parts of the input, capturing various aspects of the input information. This characteristic, along with its combination with the 1D CNN, helps enhance the accuracy of driver identification by better capturing the relationships between long-range dependencies. The 1D CNN and self-attention mechanism also address some of the limitations of traditional metaheuristic methods, such as sensitivity to initial parameter values, premature convergence, and extensive computational time. As such, they offer a more streamlined approach, with fewer control parameters and less sensitivity to their initial values.

Following the Multi-Head Self-Attention mechanism, a Global Average Pooling (GAP) layer is applied, serving as a feature descriptor by summarizing the spatial information, effectively reducing the model's output dimensionality. GAP's strategic positioning within our architecture ensures the most descriptive features are preserved, minimizing overfitting risks associated with fully connected layers. This method reduces the number of parameters by averaging the feature maps into a single value, enhancing the model's generalization ability and retaining essential spatial information for understanding time-series data such as driver behaviour patterns, unlike flattening operations. As we transition towards the final stage, a Dropout layer is incorporated to strengthen the model's generalization capabilities further. This simple yet effective regularization technique randomly nullifies a fraction of the layer's outputs during training, encouraging a more robust learning process and forcing the model to learn more generalized, noise-tolerant features. This process ensures a more reliable model for real-world, unseen data.

The final stage of the architecture comprises a fully connected layer for classification with Softmax activation, mapping the high-level features extracted from the preceding layers to individual drivers, effectively facilitating driver identification. In conclusion, the second stage of our methodology, which combines the multi-head self-attention mechanism with 1D CNN, presents a refined and efficient approach to driver identification. By leveraging the strengths of these advanced techniques, the proposed model provides a promising solution to the challenges of long-range dependency learning and time-series data processing in driver identification. By exploiting these techniques, the proposed model provides accurate and reliable driver identification, highlighting its promise in intelligent transportation systems.

The proposed model was trained using TensorFlow, employing various optimization strategies to enhance learning. Each training epoch involved random shuffling of the input samples to ensure robust learning. The backpropagation algorithm facilitated the learning process, and optimization was achieved using the Adam optimizer provided in the model compilation stage. An adaptive learning rate strategy was used, with a learning rate scheduler that reduced the learning rate when the validation loss stopped improving. The patience for the learning rate reduction was set to 5 epochs. The minimum learning rate was set to 1e-6. An early stopping mechanism was also applied to prevent overfitting and to save computational resources. This mechanism restored the best weights obtained during the training whenever the validation loss stopped improving for 20 epochs. Table 3 demonstrates 1D CNN-Attention proposed model parameter.

**Table 3:** 1D CNN-Attention proposed model parameter.

Layer	Type	Kernel size	Output channels	Padding	Stride	Other information
I0	Input	–	–	–	–	
SeparableConv1D-1	SeparableConv1D	8	128	Same	1	
BN	Batch Normalization	–	128	–	–	
Leaky-Relu	Leaky-Relu	–	128	–	–	alpha = 0.15
SeparableConv1D-2		5	64	Same	1	
BN	Batch Normalization	–	64	–	–	
Leaky-Relu	Leaky-Relu	–	64	–	–	alpha = 0.15
SeparableConv1D-3		3	32	Same	1	
BN	Batch Normalization	–	32	–	–	
Leaky-Relu	Leaky-Relu	–	32	–	–	alpha = 0.15
Attention Layer	multi_head_attention	–	32	–	–	num_heads = 2
GAP	GlobalAveragePooling1D	–	32	–	–	
Dropout	Dropout	–	32	–	–	rate = 0.2
Output	Fully connected	–	10	–	–	SoftMax

### 2.3 Cloud architecture and specification

IoT platform acts as a conduit, channelling data from a driver identification system's sensor [46] to the Google Cloud. Once on the Google Cloud, this data is stored, analyzed, and processed. This platform essentially functions as a software suite overseeing a variety of endpoints via its built-in applications.

**Fig. 9:**
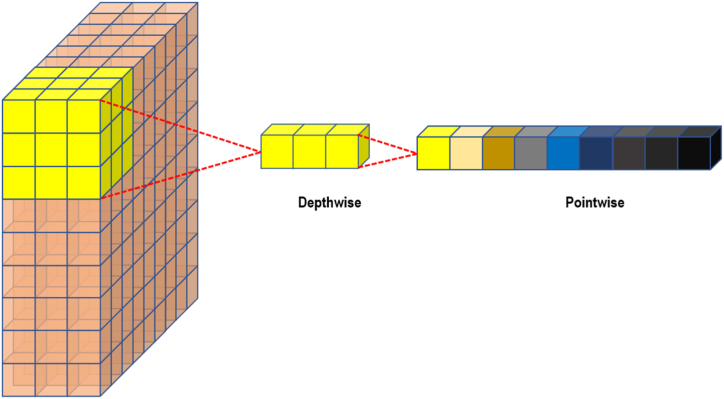
1D-separable convolution architecture.

In this context, we integrate ThingsBoard [47,48] Professional Edition with Google Cloud based on specific criteria such as the availability of an open-source solution, real-time data streaming, processing capabilities, and visualization features, as shown in Fig. 9.

**Fig. 10:**
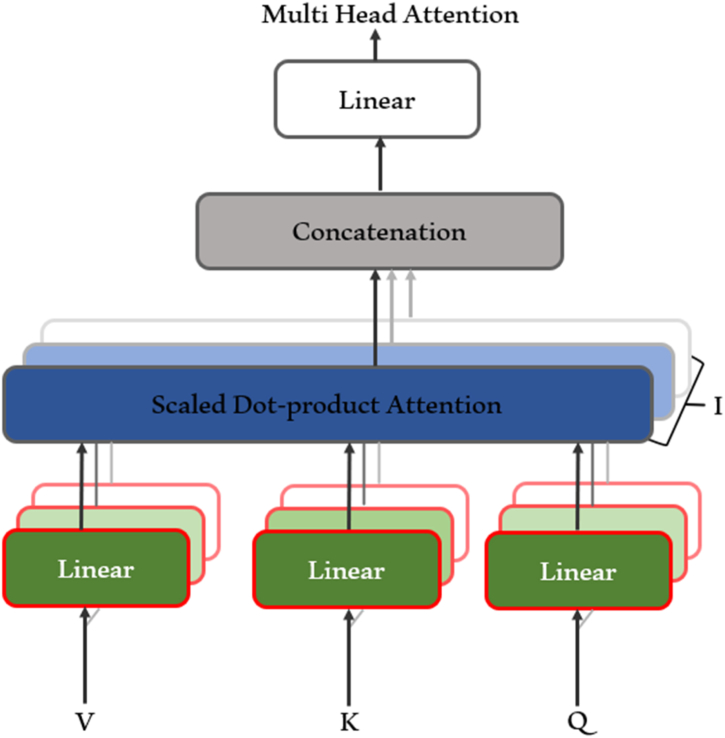
Multi-head self-attention architecture.

**Fig. 11:**
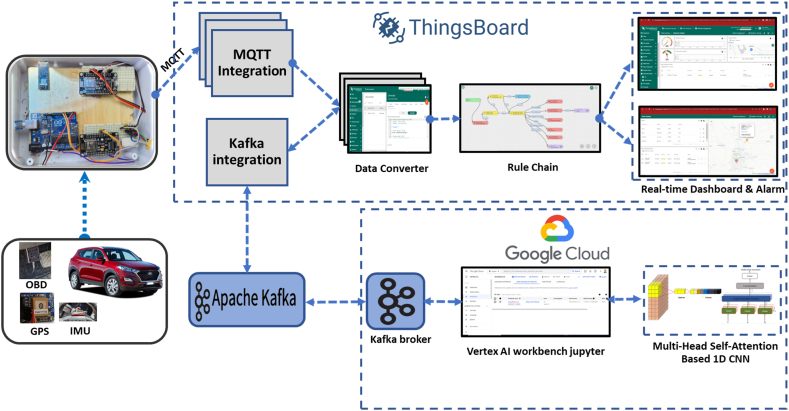
Proposed real-time driver identification architecture design.

A notable innovation in our approach is the use of Kafka for real-time DL, particularly with IoT data like OBDII, IMU, and GPS. Kafka, a distributed streaming platform, facilitates reliable and scalable data transport, ideal for the voluminous real-time data generated by IoT devices. In our configuration, a Kafka producer reads from ThingsBoard and writes to a specific Kafka topic. Subsequently, a Kafka consumer processes this and directs it to Google Cloud for real-time ML analysis. This Kafka-centric integration ensures swift, voluminous, and delay-free data transfers between ThingsBoard and Google Cloud. With this pipeline, Google Cloud can swiftly ingest and process IoT-generated data using cutting-edge ML techniques, leading to insights that can optimize the IoT system and aid in more informed decision-making.

### 2.4 Cloud-edge architecture security

Security is a critical concern when using cloud computing resources because cloud providers store and process data on behalf of their customers. In our case, security is crucial in a driver identification system based on cloud computing because it involves sensitive data, multiple users, and potential security threats. Protecting personal and sensitive data, preventing unauthorized access and system misuse, complying with legal and regulatory requirements, and maintaining system reliability and performance are essential. By implementing robust authentication, access control, monitoring, and compliance mechanisms, you can ensure the Security and Privacy of the driver identification system and mitigate the risks of security breaches and cyber-attacks.

The lack of computational power on on-field monitoring platforms can significantly challenge the implementation of robust security measures. For example, encrypting data payloads can add significant overhead to the communication process, negatively impacting the system's performance and responsiveness. Similarly, verifying the identities of users or devices may require complex algorithms and large databases, which can be computationally expensive and impractical to implement on resource-constrained devices.

To ensure the appropriate level of security in a resource-limited ecosystem of IoT devices, we can use lightweight cryptographic algorithms, implement authentication protocols optimized for IoT devices, use edge computing and gateways, and implement secure communication protocols. These measures can help fulfil security requirements such as authentication, authorization, confidentiality, and integrity of data exchange without overburdening IoT devices.

#### 2.4.1 Secure data transfer using MQTT over SSL

Although the MQTT protocol provides limited security mechanisms (e.g., username and password authentication), MQTT implementations typically utilize advanced security standards, such as SSL/TLS, to ensure secure communication at the transport layer. This provides encryption and authentication mechanisms that help to safeguard the confidentiality and integrity of data exchange between MQTT clients and brokers. Using SSL/TLS, MQTT implementations can provide a robust and secure communication channel resistant to eavesdropping, tampering, and other attacks [49]. In a system based on MQTT, the broker can authenticate the sender of a message by confirming its signature every time a message is received from a specific topic. The communication is instantly blocked and destroyed if it is not meant for that topic or comes from an unknown sender.

Furthermore, messages are encrypted with the recipient's public key to safeguard the transmitted data to maintain confidentiality. In an Edge data centre setup, messages are encrypted before being sent to the data centre (GCP); this can be accomplished by implementing access control policies, such as role-based access control (RBAC) and encryption mechanisms to protect sensitive data at rest. The results are encrypted using the data Centre's credentials before being sent to the Thingsboard platform. This ensures data is encrypted at all stages, providing a secure and robust communication channel.

#### 2.4.2 Certificate generation

All entities must have valid X.509 client authentication certificates to ensure safe and authenticated connection in a communication system comprising surveillance platforms, edge centres, and cloud layer devices. These certificates, either a public certificate created from a self-signed certificate or a root certificate issued by a certificate authority, serve as evidence of identity and authenticity. The system can enforce secure and authenticated connections, prohibit unauthorized access, and guarantee the integrity, confidentiality, and availability of transmitted data by requiring valid X.509 client certifications for all communication entities. When using a Cloud-Edges computing infrastructure, certificate generation and provisioning can be done during the setup phase since the topology of connected devices remains relatively constant. Client certificates based on the X.509 standard can be generated and installed directly on field devices and Edge computing platforms and then registered with the Cloud orchestration framework. To streamline this process, Thingsboard supports automatically configuring device X.509 credentials via its API or manual configuration via the Administrator Web UI. This approach helps ensure secure and authenticated connections between communication entities, simplifies certificate management, and enhances overall system security.

#### 2.4.3 Protocol overhead

When deploying MQTT in security-conscious environments, it's crucial to consider the protocol overhead. Unlike protocols such as HTTP, which necessitate a new connection for every request, MQTT allows clients to establish a singular connection for each session. This approach considerably reduces the overhead from frequent reconnections. However, this efficiency can be counterbalanced when implementing TLS for secure communication. While ensuring secure transmission, TLS introduces added overhead for every MQTT message. The exact overhead depends on the cypher suite used. Specifically, block cyphers can result in higher overhead due to the padding requirements compared to stream cyphers.

## 3 Performance evaluation

### 3.1 Experimental setup

Considering the importance of driver identification techniques in vehicle theft prevention and prioritizing preserving the driver's privacy, a novel method involves a model that integrates Convolutional Neural Networks (CNN) and multi-head self-attention, which operates within an end-to-end ecosystem. A cloud-based system is also developed to facilitate real-time driver identification in the context of IoV technology. To demonstrate the effectiveness of the 1D CNN-Attention, the obtained results are compared against several established techniques, including LSTM-Attention, 1D CNN, LSTM, and ANN. This comparative analysis validates the proposed approach's efficacy in driver identification. The selection of the train/test split poses two challenging concerns. Firstly, the model estimates tend to exhibit higher variance when limited training data is available. Secondly, if the testing data is insufficient, the performance statistic of the model is prone to higher variance. In general, it is crucial to carefully consider the train/test data split to ensure that neither variance becomes excessively high. This consideration is primarily influenced by the absolute number of occurrences in each group rather than just the proportion. In this study, choose an 80:20 ratio for data split.

In this study, driver identification is conducted using time series data, considering the sequential nature and interconnections between the data points. Each driver is associated with four trips, of which three are allocated for training purposes, while one is reserved for performance evaluation.

The training phase plays a crucial role in the learning process of driver identification models. During this phase, computer programs, designed as iterative learning processes, establish relationships between independent factors and dependent variables. The input and output data can be normalized to expedite this learning process, as described in equations (1), (2), (3). While the testing stage does not directly contribute to model development, it evaluates the performance of AI-based methods. The predictive performance of the model driver prediction is assessed using metrics including accuracy and macro F1 score. These measures comprehensively illustrate how well the model accurately predicts prices, as shown in the equation below.

(10) $$\text{Accuracy}=\frac{\left(\text{TP}+\text{TN}\right)}{\left(\text{TP}+\text{TN}+\text{FP}+\text{FN}\right)}$$

(11) $$Macro\;F1\;score=\frac{\sum_{i=1}^{n}{score}_{i}}{n}$$

The deep learning techniques employed in this study are comprehensively described in Table 4, Table 5, Table 6, Table 7, respectively. Based on the gathered data, the classification methods utilized for driver identification, evaluated according to various criteria, can be ranked as follows: 1D CNN-Attention > Attention-LSTM > 1D CNN > LSTM > ANN. The results presented in the tables demonstrate that the proposed approach surpasses other existing methodologies in performance.

**Table 4:** User-defined parameter for the applied method.

Method	Parameters
LSTM-Attention	Two attention heads
LSTM	Five layers, 30 neurons
1D CNN	Five layers, 30 neurons
ANN	Two layers, 30 filters

**Table 5:** The accuracy and Macro F1 score analysis for the proposed models across different window size.

Window time - Overlap (40-20)						
Performance metrics		Model				
		ANN	LSTM	CNN	LSTM-Attention	1D CNN-Attention
**Accuracy**	**Training**	77.02%	94.19%	94.03%	97.90%	98.31%
	**Testing**	87.98%	99.17%	99.11%	99.19%	99.62%
**F1-Score**	**Training**	76.30%	93.89%	93.81%	97.72%	98.21%
	**Testing**	87.84%	99.13%	99.08%	99.18%	99.60%
**Window time - Overlap (40**–**30)**						
**Performance metrics**		ANN	LSTM	CNN	LSTM-Attention	1D CNN-Attention
**Accuracy**	**Testing**	84.45%	98.62%	99.03%	99.19%	99.78%
	**Training**	94.16%	99.41%	99.57%	99.57%	99.69%
**F1-Score**	**Testing**	84.09%	98.85%	99.00%	99.13%	99.77%
	**Testing**	93.92%	99.38%	99.57%	99.57%	99.67%
**Window time - Overlap (60**–**30)**						
**Performance metrics**		ANN	LSTM	CNN	LSTM-Attention	1D CNN-Attention
**Accuracy**	**Testing**	80.68%	95.09%	96.43%	97.41%	98.70%
	**Training**	86.37%	98.90%	99.27%	99.87%	99.72%
**F1-Score**	**Testing**	80.05%	94.91%	96.40%	97.28%	98.63%
	**Testing**	85.54%	99.00%	99.21%	99.85%	99.70%
**Window time - Overlap (60**–**40)**						
**Performance metrics**		ANN	LSTM	CNN	LSTM-Attention	1D CNN-Attention
**Accuracy**	**Testing**	73.35%	92.96%	94.47%	96.97%	98.81%
	**Training**	92.11%	99.08%	98.18%	99.70%	99.78%
**F1-Score**	**Testing**	73.30%	93.09%	94.44%	97.11%	98.83%
	**Testing**	91.93%	99.02%	98.13%	99.68%	99.77%
**Window time- Overlap (60**–**50)**						
**Performance metrics**		ANN	LSTM	CNN	LSTM-Attention	1D CNN-Attention
**Accuracy**	**Testing**	83.22%	96.90%	98.26%	99.13%	99.95%
	**Training**	92.04%	99.21%	99.21%	99.70%	99.99%
**F1-Score**	**Testing**	82.45%	96.87%	98.12%	99.12%	99.95%
	**Testing**	91.78%	99.21%	99.21%	99.68%	99.99%

**Table 6:** Assessing performance in the presence of anomalous data.

Anomaly Rate	Anomaly Duration	Accuracy with Anomalies				
		ANN (%)	LSTM (%)	CNN (%)	LSTM- Attention (%)	1D CNN-Attention (%)
0%	1s	80.68	94.91	96.43	97.74	**98.7**
	10s	80.68	94.91	96.43	97.74	**98.7**
1%	1s	80.57	94.33	96.43	97.24	**98.82**
	10s	80.62	94.33	96.43	97.24	**98.82**
10%	1s	79.87	91.25	92.71	93.19	**97.47**
	10s	79.44	90.92	92.06	93.35	**97.58**
30%	1s	77.23	85.25	87.08	90.76	**94.62**
	10s	77.07	85.25	87.14	88.49	**94.56**
50%	1s	75.73	77.31	84.82	90.92	**92.03**
	10s	75.46	77.8	85.04	88.01	**91.87**

**Table 7:** Performance evaluation of corrected data.

Anomaly Rate	Anomaly Duration	Corrected Anomalies (One-Class SVM)				
		ANN (%)	LSTM (%)	CNN (%)	LSTM- Attention (%)	1D CNN-Attention (%)
0%	1s	80.68	94.91	96.43	97.74	**98.7**
	10s					
1%	1s	80.01	94.62	96.08	97.72	**98.91**
	10s					
10%	1s	79.79	93.7	94.08	96.63	**97.5**
	10s					
30%	1s	78.87	92.78	95.33	95.65	**97.39**
	10s					
50%	1s	77.46	92.23	93.64	94.89	**96.2**
	10s					

In our experimental configuration, we employed two platforms. The Google Cloud Platform was utilized for deep learning tasks, while Thingsboard was the visualization tool for result analysis. The virtual machine used in the experiments was equipped with four virtual CPUs, 15 GB of RAM, and a TensorFlow 2.11 environment. A Kafka server was also implemented to facilitate data streaming between Thingsboard and Google Cloud, ensuring seamless bidirectional communication.

### 3.2 Results and discussion

Moreover, the 1D CNN-Attention algorithm was identified as being the most efficient approach for classifying driver behaviour. The proposed technique demonstrated superior metric performance, including accuracy and F1-score, as well as significantly outperformed other algorithms in terms of computational time. These findings highlight the superiority of the proposed algorithm over alternative methods. The results of the five ML methods (ANN, CNN, LSTM, LSTM with Attention, and 1D CNN-Attention) were evaluated based on accuracy and F1-score metrics for five different window sizes with overlap (40-20, 40-30, 60-30, 60-40, and 60-50). Among these models, it was observed that the CNN with attention model consistently outperformed the other models. The F1 score considers the balance between precision (the ability to identify positive instances correctly) and recall (the ability to identify all positive instances correctly). The higher F1-score obtained by the 1D CNN-Attention model indicates its effectiveness in achieving a balanced classification performance due to its feature extraction capabilities, attention mechanisms for improved focus on essential features, handling of sequential dependencies, and robust representation learning. As shown in Table 5, the multi-head attention-based 1-D separable CNN model demonstrates superior performance compared to other models in the context of the Security Driveset dataset. It achieves impressive scores ranging from 99.62 % to 99.99 % across different window sizes. The experiment revealed that the best results were obtained when using a window size of 60 s with a high degree of overlap. Specifically, a shift value of dx = 10 was employed, resulting in a 50 s overlap (60 - 10 = 50).

**Fig. 12:**
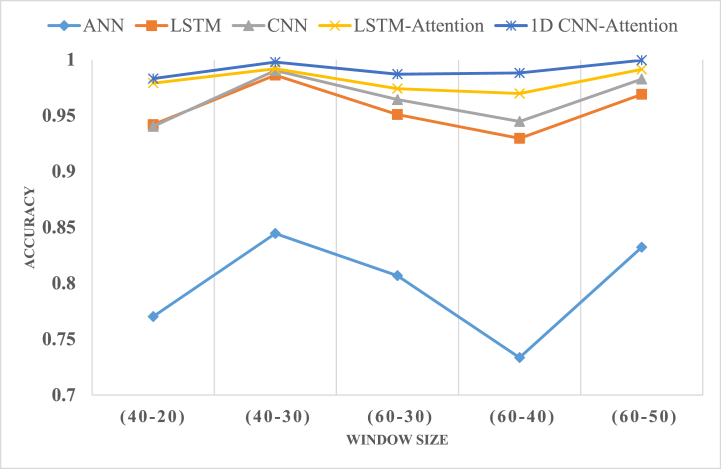
Performance evaluation of different models for five window sizes.

An important observation to consider is that while increasing the degree of overlap does not impact the network size, it does have an effect on the model's performance. This relationship is illustrated in Fig. 12, where different window size configurations are examined. In the first point of the graph, a window size of 40 s is utilized with an overlap of 20 s, resulting in a 50% overlap. Notably, the accuracy of the model increases. Moving to the second point, the overlap is further increased to 30 s while maintaining a window size of 40 s. The increase in overlap leads to a noticeable improvement in accuracy. However, despite increasing the window size to 60 s at the third point, the overlap is reduced to 30 s. Surprisingly, this adjustment results in a decrease in accuracy. The trend continues in the fourth and fifth points, where the overlap is increased to 40,50 s, respectively, while maintaining a window size of 60 s.

These findings highlight the significance of selecting appropriate window sizes and overlap values to achieve optimal performance for the given task. The results suggest that a larger window size and a higher degree of overlap can improve the model's effectiveness in capturing relevant patterns and dependencies within the data.

**Fig. 13:**
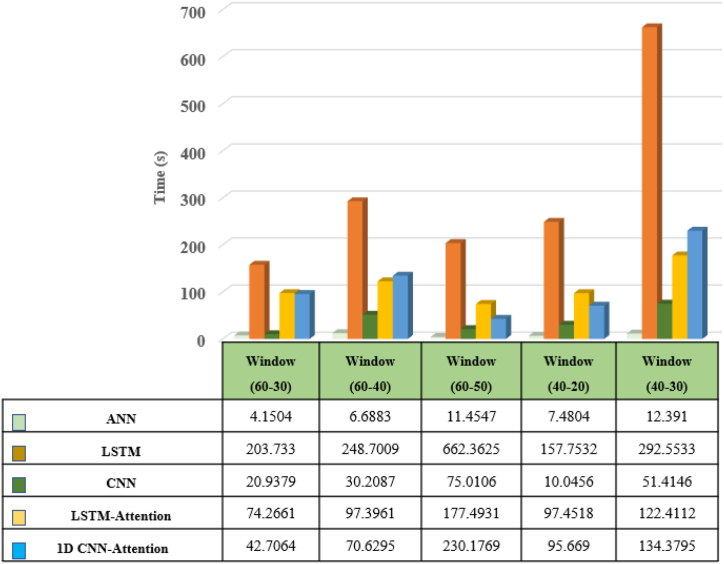
Visual comparison of the proposed 1-D CNN Attention model with different models in terms of training time for five different window sizes.

The inference time results for different window sizes and overlaps are presented, summarizing the training and testing computational times (in seconds) in Fig. 13. Notably, the CNN-based models, namely CNN and 1D CNN-Attention, demonstrate superior computational efficiency compared to the LSTM-based models (LSTM and LSTM with Attention). While the ANN model exhibits relatively shorter computational times, it may have limitations in capturing complex patterns compared to deep learning models. It is important to consider that the computational efficiency of each model can vary depending on factors such as window size, overlap, dataset characteristics, and hardware configurations. These factors play a crucial role in determining the specific computational performance of each model. Overall, the results highlight the advantages of CNN-based models regarding computational efficiency while noting the trade-off between computational speed and the model's ability to capture intricate patterns.

### 3.3 Robustness to abnormal data patterns (data anomalies)

Driver identification accuracy relies on the dependable nature of vehicle sensor data patterns. However, Sensors are prone to errors, malfunctions, and even hacking attempts. Due to this vulnerability, the model may be fed with erroneous and aberrant data, producing false predictions. To address this issue, a driver identification system should include mechanisms capable of effectively detecting and correcting anomalies. By incorporating such mechanisms, the system can ensure accurate predictions and maintain its performance even in the presence of anomalous data. Considering this challenge, a proposed robustness analysis aims to evaluate the performance of the proposed classification model when anomalies are present. This analysis seeks to assess the model's ability to maintain accurate predictions even in the presence of anomalous data.

We adopted the same experimental settings presented in Ref. [8] to guarantee an equitable comparison between our suggested and earlier models. In this study, we evaluated One-Class Support Vector Machine (SVM) models for anomaly detection in driver identification. The models were trained individually for each sensor using fixed-size windows of 5 s. A sliding window approach with a duration of 1 s was employed for training and evaluation. The performance of the models was assessed based on the accuracy of 1-D CNN Attention after anomaly detection. To address the correction of anomalies, we employed a strategy of using the average value from the closest timestamps. Specifically, the previous and the following timestamp values were considered, and their average was used to correct the detected anomalies.

The analysis utilized the Security Driving Dataset for experimentation purposes. Specifically, a subset of the dataset consisting of 311 data records was selected for analysis. Each record in the dataset contained numerical values corresponding to a 60-s driving duration. The dataset comprised measurements from 15 different sensors, which served as features for the analysis. A random selection of n sensors where n equals seven, as shown in Table 6, was chosen to introduce anomalies into the dataset. For these selected sensors, incorrect random values were injected to simulate anomalous conditions. By employing this approach, the analysis aimed to evaluate the performance of the proposed model under the presence of intentionally introduced anomalies.

To assess the impact of varied data anomaly rates, we conducted simulations and evaluated the accuracy of the 1D CNN-Attention model with and without anomaly correction. Table 6, Table 7 provide the findings of these assessments. We specifically focus on the accuracy of the 1D CNN-Attention model in the presence of various anomaly rates, ranging from 0% to 50%. Moreover, the anomalies' duration was also considered during the evaluation. We conducted simulations for each anomaly rate, considering two different anomaly durations: 1 s and 10 s. An anomaly duration of 10 s implies that, for each modified record, 10 of the total 60 values (the time length of the record) were modified. The results of these simulations are presented in Table 6. It is observed that, particularly in the case of a 50% anomaly rate, the model's accuracy dropped. This indicates the adverse impact of a higher anomaly rate on the model's performance.

Table 7 compares the accuracies achieved by five different models (ANN, CNN, LSTM, LSTM Attention, and 1D CNN-Attention) after correcting anomalies detected using the one-class SVM model. The results demonstrate that, in most cases, anomaly detection led to improved accuracy. However, regardless of whether anomaly detection was used, the 1D CNN-Attention model consistently outperformed the other models. This is evident in both Table 6, Table 7. For instance, in the case of a 50% anomaly rate, the accuracy dropped from 99.99 to 91.87 when anomaly correction was not applied. However, when the one-class SVM model was used for anomaly detection, the accuracy improved to 96.2. These results highlight the effectiveness of the 1D CNN-Attention model, as it demonstrates superior performance even in challenging scenarios with a high anomaly rate.

### 3.4 Statistical analysis using the Friedman test

To demonstrate a significant distinction between the proposed model (1D CNN-Attention) and other ML, single and hybrid DL models within various window sizes, a two-step statistical testing approach was employed for method validation. Following the recommendations of Demšar [51], an extensive analysis was performed using the Friedman rank test as the initial step. Friedman post hoc testing was performed if variations in regressor performances were found. The Iman-Davenport test assessed whether any model showed a substantial advantage over the others, while the Friedman test was used to construct the hierarchy of benchmarked models. Subsequently, upon detecting a difference, a pairwise test using the Friedman post hoc test with the corresponding p-value was carried out for multiple comparisons.

The Friedman post hoc test was used to compare the other models to the reference model, the proposed model (1D CNN-Attention), which served as the standard of comparison. A p-value below the threshold of 0.05 was used to determine the significance level of differences. Table 8 presents the p-value and the average rank obtained from Friedman's Iman-Davenport test. Remembering that a model's more outstanding excellence equates to a lower rank is crucial. The 1D CNN-Attention method is the best-performing model according to the data in Table 8 since it has the lowest rank. A statistically significant difference (p-value less than 0.05) among at least two benchmarked techniques is shown by the p-value of 0.0006678. As a result, we can rule out the null hypothesis that all models perform similarly.

**Table 8:** Outcomes of each model's Iman-Davenport tests and Friedman rank.

Model	Friedman Ranks	Iman Davenport p-value
Artificial Neural Network (ANN)	625	0.0006678
Long Short-Term Memory (LSTM)	361	
Convolution Neural Network (CNN)	256	
LSTM-Attention	100	
1D CNN-Attention	25	

Moreover, after the null hypothesis was rejected, the Friedman post hoc test was used to assess each pairwise combination's performance. The results of the statistical comparison for the pairs are presented in Table 9. Remarkably, there were statistically significant performance differences (p-value <0.05) between the proposed model (1D CNN-attention) and all the existing models. These findings indicate that the 1D CNN-Attention model exhibits superior learning capabilities compared to the other models.

**Table 9:** The Friedman post hoc test results for all models related to the suggested model (1D CNN-Attention).

Comparison	Post-hoc p-value
ANN VS 1D CNN-Attention	1.37839E-05
LSTM1D VS CNN-Attention	0.009540951
CNN VS 1D CNN-Attention	0.033868306
LSTM-Attention VS 1D CNN-Attention	0.043179751

### 3.5 Comparative analysis

This study conducted a comparative analysis to evaluate and compare various models, techniques, or approaches employed in driver identification. The objective was to assess their performance and efficacy in addressing the challenges associated with driver identification. The findings are presented in Table 10, which provides a comprehensive overview of the latest advancements in driver identification techniques reported in recent literature. Among the listed models, the proposed 1D CNN-Attention model exhibited outstanding performance in the security dataset, achieving the highest accuracy and F1-score of 0.9995. This model outperformed other approaches by a significant margin, indicating its superior performance in accurately identifying drivers. Furthermore, the proposed 1D CNN-Attention model demonstrated remarkable efficiency in model training, with a short training time of 230 s. In contrast, models such as Hybrid depth-wise CONV-LSTM/GRU and Hybrid FCN-LSTM with self-attention required significantly longer training durations of 833 min and 308 min, respectively. Comparatively, references such as Ezzini et al. [27], Kwak et al. [32], El Mekki et al. [40], Azadani et al. [43], and Hongyu et al. [52] achieved accuracy values of 96.20%, 98.06%, 95.1%, 95.03%, and 95.27%, respectively. However, these references did not mention the corresponding F1 score or training times. On the other hand, references such as Abdennour et al. [37], Shan et al. [38], Zhang et al. [39], Azadani et al. [42], and Abu-gellban et al. [45] reported accuracy values of 99.3%, 98.72%, 97.01%, 95.06%, and 90%, respectively. In conclusion, the proposed 1D CNN-Attention model emerged as the top-performing approach, achieving the highest accuracy and F1 score while exhibiting efficient training times. These results underscore the effectiveness of this model in accurately classifying driver identification datasets.

**Table 10:** Comparative table for driver identification classification in terms of Accuracy, Macro F1-score, and training time.

Reference	Year	Model	Dataset	Accuracy (%)	F1-score (%)	Training time
Proposed	present	1D CNN-Attention	Security dataset	0.9995	0.9995	230 s
Ezzini et al. [27]	2018	Extra tree		0.9620	–	–
Kwak et al. [32]	2020	SVM		0.9806	–	–
Girma et al. [36]	2019	LSTM		–	0.98	–
Abdennour et al. [37]	2021	Deep RCN		0.993	0.9933	108 min
Shan et al. [38]	2020	Hybrid depth-wise CONV-LSTM/GRU		0.9872	–	833 min
Zhang et al. [39]	2019	Hybrid FCN-LSTM with self-attention		0.9701	0.9702	308 min
El Mekki et al. [40]	2019	Hybrid FCN-LSTM		0.951	–	–
Azadani et al. [42]	2020	Hybrid 1D–CNN–BLSTM		0.9506	0.9492	–
Azadani et al. [43]	2021	Hybrid DeepConvLSTM		0.9503	–	–
Abu-gellban et al. [45]	2020	LiveDI		0.90	0.86	–
Hongyu et al. [44]	2021	1-D CNN		–	89.9	–
Hongyu et al. [52]	2023	Ensemble M 1-D CNN with BLSTM		0.9527	–	–
**Collected Dataset**						
Del et al. [28]	2014	ANN	brake pedal pressure and gas pedal pressure	0.846	–	–
Zhang et al. [29]	2016	SVM	Throttle position B, Gyroscope Torque GPS Accelerometer, Acc. pedal position E, Engine RPM, Acc. Pedal position D Throttle position manifold, Relative throttle position	0.9322	–	–
Martinez et al. [30]	2018	Extreme-learning machine core	UYANIK in Turkey	0.75	–	–
Hallac et al. [33]	2016	RF	Engine RPM, Gas pedal position, Torque, Steering velocity, Vehicle velocity, Steering wheel angle, Lateral acceleration, Vehicle heading, Brake pedal position, Forward acceleration, Throttle position, Steering acceleration.	0.501	–	–
Jong et al. [35]	2018	CNN	Steering wheel rotation speed, Longitudinal acceleration, Brake pedal pressure, Accelerator pedal pressure, Vehicle Speed, Engine RPM, Lateral acceleration (Lat-Accel), Yawrate, Air blower, Dynamic traction control	0.90	–	–

On the other hand, Table 11 provided compares three models: FCN-LSTM, Deep Conv-GRU, and the proposed 1D CNN-Attention. The models are evaluated based on their accuracy with different anomaly rates and durations. The accuracy after applying the correction method of One-Class SVM is also included for comparison. Our proposed 1D CNN-Attention model generally performs slightly better than the other two models, with accuracy scores above 98% for most cases. This suggests that the attention mechanism implemented in the proposed model helps capture relevant information and improve anomaly detection. After applying the correction method of One-Class SVM, the accuracy improves, indicating that the One-Class SVM algorithm effectively corrects anomalous predictions. However, the proposed 1D CNN-Attention model consistently demonstrates the highest accuracy scores after correction, outperforming the other two models.

**Table 11:** Comparative table in terms of accuracy in the presence of anomalous data.

Anomaly Rate	Anomaly Duration	Accuracy with Anomalies			Corrected Anomalies (One-Class SVM)		
		Proposed 1D CNN-Attention	FCN-LSTM	Deep Conv-GRU	Proposed 1D CNN-Attention	FCN-LSTM	Deep Conv-GRU
0%	1s	98.7%	95.1%	98.72%	98.7%	95.1%	98.72%
	10s	98.7%	95.1%	98.72%			
1%	1s	98.82%	93.25%	98.08%	98.91%	93.89%	97.32%
	10s	98.82%	92.6%	98.08%			
10%	1s	97.47%	85.85%	92.74%	97.5%	93.57%	97.12%
	10s	97.58%	84.89%	93.16%			
30%	1s	94.62%	70.42%	82.69%	97.39%	92.93%	96.32%
	10s	94.56%	69.77%	81.2%			
50%	1s	92.03%	57.23%	72.86%	**96.2**%	91.64%	95.14%
	10s	91.87%	57.88%	75.21%			

### 3.6 Model performance on collected data

We conducted a performance evaluation by measuring the accuracy of our proposed base learners and comparing them with other state-of-the-art ML methods such as ANN, CNN, LSTM, and LSTM-attention for time series data classification. The performance metrics (accuracy and F1-score) for five different deep learning models, ANN, LSTM, CNN, LSTM-Attention, and 1D CNN- Attention, are present in Table 12. The models are evaluated using different window size configurations: Overlap (40-30), Overlap (60-30), Overlap (60-40), and Overlap (60-50). Each experiment was repeated five times to ensure the robustness of the results. Throughout all epochs, the 1-D CNN attention model consistently performed more than the other models. It consistently achieved higher accuracy scores, indicating its effectiveness in classifying time series data. Following closely behind, the LSTM-attention model demonstrated the second-best performance, surpassing both the LSTM and CNN models. The results of LSTM-attention were slightly better than those of LSTM and CNN, showcasing the added benefit of incorporating attention mechanisms.

**Table 12:** The accuracy and Macro F1 score analysis for the proposed models across different window sizes for collected data.

Window size- Overlap (40-20)						
Performance metrics		Model				
		ANN	LSTM	CNN	LSTM- Attention	1D CNN-Attention
Accuracy	Training	93.12%	94.74%	95.79%	96.83%	97.89%
	Testing	88.42%	97.62%	98.41%	98.41%	99.74%
F1-Score	Training	92.12%	94.25%	95.35%	96.13%	97.67%
	Testing	88.17%	97.07%	98.05%	98.04%	99.68%
**Window size - Overlap (40**–**30)**						
Accuracy	Training	91.95%	95.76%	98.31%	99.15%	99.15%
	Testing	94.07%	96.19%	98.31%	99.79%	99.79%
F1-Score	Training	90.91%	93.83%	97.50%	98.80%	98.80%
	Testing	91.57%	95.57%	98.06%	99.76%	99.76%
**Window size - Overlap (60**–**30)**						
Accuracy	Training	92.86%	94.62%	98.21%	97.87%	98.21%
	Testing	97.84%	98.65%	98.65%	98.38%	99.55%
F1-Score	Training	90.48%	90.30%	97.67%	97.30%	97.78%
	Testing	97.37%	98.41%	98.41%	98.04%	99.47%
**Window size - Overlap (60**–**40)**						
Accuracy	Training	91.43%	94.29%	95.71%	97.14%	98.57%
	Testing	93.50%	99.28%	99.64%	99.64%	100.00%
F1-Score	Training	89.29%	92.31%	94.34%	96.30%	98.11%
	Testing	92.86%	99.15%	99.58%	99.57%	100.00%
**Window size - Overlap (60**–**50)**						
Accuracy	Training	93.48%	96.38%	97.10%	98.55%	99.28%
	Testing	98.19%	98.00%	99.46%	99.09%	99.64%
F1-Score	Training	92.04%	95.58%	96.43%	98.25%	99.13%
	Testing	97.79%	97.55%	99.34%	98.90%	99.57%

### 3.7 Implementation within Thingsboard and Google Cloud

**Fig. 14:**
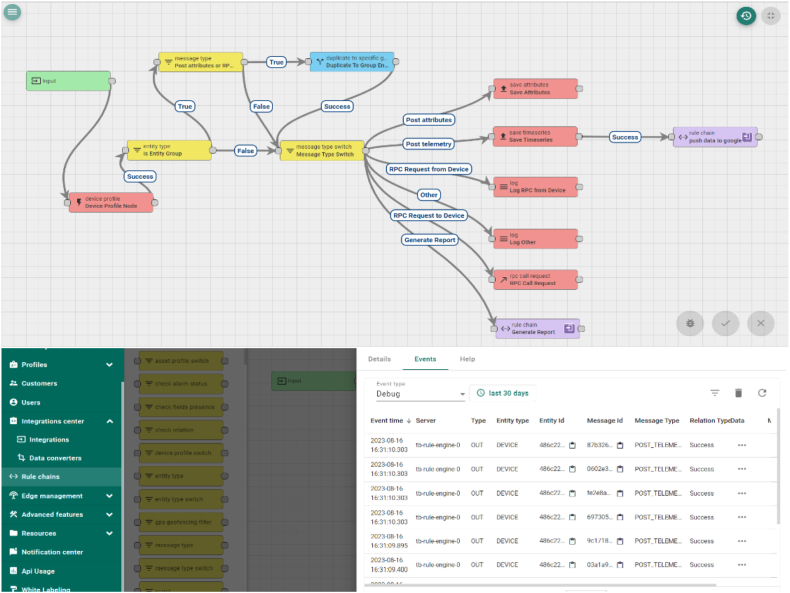
Thingsboard Rule chain stream data from Nodemcu to google cloud.

ThingsBoard is an open-source IoT platform designed for comprehensive device management, efficient data collection, seamless processing, and intuitive visualization for IoT devices. However, the platform has inherent processing limitations restrict its ability to perform advanced analytics. Although ThingsBoard offers basic analysis features, such as threshold crossing, it lacks support for more sophisticated analytics like ML and predictive analytics. This limitation necessitates the utilization of a rule chain within ThingsBoard.

to intelligently route data from IoT. This limitation necessitates utilizing a rule chain within ThingsBoard to intelligently route data from IoT devices to diverse plugins based on device attributes or the data itself. To address the need for advanced analytics, we propose hosting a 1D CNN-Attention model on a Google Cloud instance with ample resources, including 4vCPU and 15 RAM. This robust infrastructure will facilitate the execution of complex analytical tasks. Fig. 14 presents a seamless data flow; telemetry data from the edge device (NodeMCU) is transferred to the Thingsboard device via a secure Wi-Fi connection. Once within the Thingsboard ecosystem, the rule chain comes into play, enabling rudimentary analysis.

**Fig. 15:**
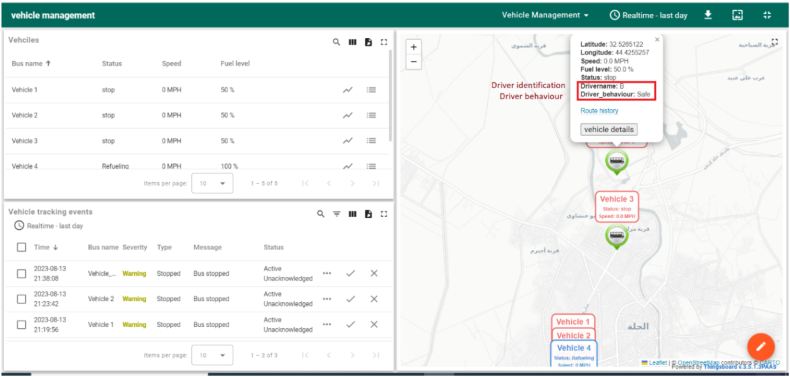
Vehicle management dashboard.

To ensure efficient and reliable data transfer, a Kafka server is a robust intermediary for transmitting data between ThingsBoard and the Google Cloud infrastructure. This Kafka server acts as a conduit for transferring data from ThingsBoard to the Google Cloud platform for processing and returning the processed results to ThingsBoard for visualization purposes, as shown in Fig. 15. The trained model, developed using the collected data, is deployed and hosted on the Google Cloud platform. Its primary objective is to perform driver identification classification tasks. Leveraging the extensive capabilities of the Google Cloud infrastructure, including ample computational resources, the model can execute sophisticated analytics and deliver accurate driver identification outcomes.

Through the seamless integration of the Google Cloud Platform and ThingsBoard, this proposed architecture enables the deployment of the trained model to conduct advanced analytics within the broader IoT solution. The architecture maintains the driver (Driver B) within the ThingsBoard ecosystem, preserving its association with the platform's functionalities. This integration offers significant advantages by harnessing the analytical capabilities of the trained model and combining them with the comprehensive features of ThingsBoard. The result is an enhanced decision-making process and the generation of valuable insights for real-life use cases, ultimately empowering efficient and effective driver identification within the IoT environment.

## 4 Conclusion

This paper presents an innovative method for real-time driver identification using a cloud computing system integrated with a deep learning model. The proposed system showcases a remarkable integration between Google Cloud, Thingsboard, and Apache Kafka, leveraging the robust infrastructure and scalable processing capabilities of Google Cloud. This comprehensive solution enables seamless real-time data collection, processing, prediction, and visualization designed for the IoV technology. The system demonstrates its effectiveness and reliability by successfully addressing various real-world scenarios, including sensor data anomalies. The study emphasizes the significance of the proposed approach, highlighting its potential for practical applications in the field of IoV. In addition, this paper develops and validates an interpretable multi-head self-attention and CNN-based framework deployed on Google Cloud for accurate driver identification. The evaluation uses two practical datasets: the security and the collected datasets. Performance metrics, including accuracy and Macro F1 score, are employed to assess the effectiveness of the models in different scenarios. The results demonstrate that the proposed 1DCNN-Attention model outperforms alternative models, including ANN, CNN, LSTM, and LSTM-Attention. The 1D CNN-Attention model achieves the highest accuracy and Macro F1 score with the lowest training time for a 60-s window size and 10-step time driver identification classification on the practical datasets. Compared to different approaches, the proposed 1D CNN-Attention has the highest (accuracy and Macro F1 score) and low training time for a 60-s window size and 10-step driver identification classification for two practical data sets. For instance, in the case of the public data set (security data set), the highest accuracy, Macro F1 score, and lowest training time values were in a 60-s window size of 99.95%, 99.95%, and 230 s, respectively. In the case of data anomaly, the highest accuracy value was achieved for window size 60 s and step size 30 s, which was 91% in the case of the highest anomaly rate of 50%. Finally, when using the anomaly-corrected algorithm, the highest accuracy for window size is 60 s, and step size is 10 s, 96% in case of a 50 % anomaly rate.

However, it is important to note some limitations of this work. The study focuses on a single public dataset (Security dataset). It only involves four drivers in the collected dataset, limiting the findings' generalizability to other datasets. Additionally, the proposed model requires substantial data for effective learning. To address these limitations, future extensions of this work should include testing the proposed method on different datasets and routes to enhance generalizability. Moreover, employing few-shot learning techniques can improve system efficiency and reduce the data required for effective learning.

The practical and social implications of this study are significant. The developed model can be implemented in the General Traffic Directorate to improve fleet management, driver profiling, vehicle security, personalized driving experiences, insurance and risk assessment, and road safety. By accurately identifying drivers in real-time, the model contributes to enhancing overall traffic safety and security, while also providing personalized and efficient driving experiences. Furthermore, insurance companies can use the model to assess driver behaviour and determine appropriate insurance premiums, promoting responsible driving habits. Overall, this research has practical implications for the automotive industry and social implications for road safety and transportation management.

## CRediT authorship contribution statement

**Hassan Muwafaq Gheni:** Project administration, Methodology, Data curation. **Laith A. AbdulRahaim:** Visualization, Validation, Supervision. **Abdallah Abdellatif:** Writing – review & editing, Software.

## Declaration of competing interest

The authors declare that they have no known competing financial interests or personal relationships that could have appeared to influence the work reported in this paper.
